# Supplementary material for: Changing measurements or changing movements? Sampling scale and movement model identifiability across generations of biologging technology
Source: Ecol Evol. 2017 Oct 3;7(22):9257–66. doi: 10.1002/ece3.3461 (PMC5696428; doi:10.1002/ece3.3461)
Supplement: Supplementary file 1 [file ECE3-7-9257-s001.pdf]

Supplemental Appendices for: Changing measurements or changing movements? Sampling scale and movement model identifiability across generations of biologging technology

Leah R. Johnson[1,4*], Philipp H. Boersch-Supan[2,3,4], Richard A. Phillips[5], and Sadie J. Ryan[2,3]

[1]Department of Statistics, Virginia Tech, Blacksburg, VA 24061, USA
[2]Department of Geography, University of Florida, Gainesville, FL 32601, USA
[3]Emerging Pathogens Institute, University of Florida, Gainesville, FL 32610, USA
[4]Department of Integrative Biology, University of South Florida, Tampa, FL, USA
[5]British Antarctic Survey, Natural Environment Research Council, Cambridge, CB3 0ET, UK
*lrjohn@vt.edu

September 25, 2017

Running Head: Sampling scale and movement model identifiability

S.1 Parsing and cleaning of data

Immersion data (wet/dry records) were downloaded in the field and stored in a variety of file formats. We developed a suite of parsing functions to import these records into R and format the data for further analysis. Cleaned data and code for the data analysis will be deposited in the Dryad online repository (`https://datadryad.org/`).

Flight length calculations

Wet/dry records were parsed at the highest temporal resolution for each type of logging device, before flight lengths were calculated by merging consecutive time periods recorded as dry. In line with previous studies (Edwards et al., 2007), only dry segments with a

duration over 30 seconds were counted as flights, to avoid counting preening events and other behaviors that might involve the leg with the logger extended out of the water.

S.2 Distributions for step lengths

We compare 4 distributional models for flight times: shifted exponential, pareto, shifted gamma (which is related to an exponentially truncated pareto), and the shifted q-exponential. Below we give brief details on each of these distributions. Implementations of these distributions in R are included in the supplementary materials.

Shifted Exponential Distribution

The shifted exponential is a generalization of the exponential where the support has been shifted some positive amount x_0. That is the pdf, $f(x)$ is given by

$$f(x) = \begin{cases} 0, & \text{for } x < x_0 \\ \lambda e^{-\lambda(x-x_0)} & \text{for } x \geq x_0 \end{cases} \tag{1}$$

where $\lambda > 0$ is the rate parameter. Similarly was can say that $X - x_0 \sim \text{Exp}(\lambda)$. The theoretical mean of the exponential is $1/\lambda$. If the data were observed directly, the maximum likelihood estimator (MLE) is given by

$$\hat{\lambda} = \frac{1}{\bar{x} - x_0} \tag{2}$$

where $\bar{x}$ is the sample mean.

Pareto Distribution

The Pareto (Type 1) distribution is is power law probability distribution defined above a lower limit, x_0. The pdf, $f(x)$ is given by:

$$f(x) = \begin{cases} 0, & \text{for } x < x_0 \\ \frac{\alpha x_0^\alpha}{x^{\alpha+1}} & \text{for } x \geq x_0 \end{cases} \tag{3}$$

where $x_0 > 0$ is the minimum possible value (that also determines the scale of the process), and $\alpha > 0$ is a unitless shape parameter. This formulation is the same as the typical power law used in Levy studies, but with the shape parameter redefined so that $\alpha = \mu - 1$ so that the parameter is constrained to be positive instead of > 1. The mean and variance of the Pareto are only defined for a subset of parameter values: for $\alpha < 1$ the mean approaches infinity, and the variance is also infinite for $\alpha \leq 2$.

In this paper, we assume that we have a biologically defined lower limit, x_0, so we are only concerned with estimating the shape parameter, α. If the data were observed without error, the MLE for α is given by

$$\hat{\alpha} = \frac{n}{\sum_i (\ln x_i - \ln x_0)} \tag{4}$$

where n is the number of data points. If we were also estimating x_0, the MLE is simply $\hat{x_0} = \min_i x_i$, and this estimator would be plugged into Eqn. 4.

Shifted Gamma Distribution

The shifted gamma distribution is a generalization of the gamma where the support has been shifted some positive amount x_0. That is the pdf, $f(x)$ is given by

$$f(x) = \begin{cases} 0, & \text{for } x < x_0 \\ \frac{\beta^\alpha}{\Gamma(\alpha)}(x - x_0)^{\alpha-1} e^{-\beta(x-x_0)} & \text{for } (x) \geq x_0. \end{cases} \tag{5}$$

where $\alpha > 0$ is the shape parameter and $\beta > 0$ is the rate parameter. Similarly was can say that $X - x_0 \sim \text{Gamma}(\alpha, \beta)$. The theoretical mean of the standard Gamma distribution

in this case is given by α/β and the variance by α/β^2. There are no closed form MLEs for both parameters of the Gamma distribution. Instead we find these numerically.

Shifted Q-Exponential Distribution

The q-exponential distribution is a generalization of the exponential distribution with heavy (possibly power-law) tails. The pdf, $f(x)$ of the shifted distribution is given by

$$f(x) = \begin{cases} 0 & \text{for } x < x_0 \\ (2-q)\lambda e_q(\lambda(x - x0)) & \text{for } (x) \geq x_0. \end{cases} \tag{6}$$

where $1 \leq q < 2$ is the shape parameter, $\lambda > 0$ is the rate parameter and

$$e_q(x) = [1 + (1-q)x]^{\frac{1}{1-q}}.$$

When $q = 1$ we regain the exponential distribution. Similarly was can say that $X - x_0 \sim$ Pareto(q, λ). Here we restrict ourselves to the case where $1 \leq q < 2$ as this is the range of q for which the support of the distribution is on $[0; \infty)$. As with the gamma, we determine the MLE for the parameters analytically.

S.3 Multinomial likelihoods for the the biologger data

As in Edwards et al. (2007), we use a multinomial maximum likelihood approach to estimate the parameters of the underlying flight process while taking into account the observational process of the biologgers. Here we briefly present the likelihoods described in detail in the Supplementary Materials 1 and 2 from Edwards et al. (2007), with the equations generalized slightly to account for differences in sampling protocols.

Most generally, the log-likelihood of the PDF parameters $\boldsymbol{\theta}$, given a set of observations $\mathbf{r}$ take the general form

$$\ell(\boldsymbol{\theta}|\mathbf{r}) = \sum_{j=1}^{J} d_j \log[P(j|\boldsymbol{\theta})] \tag{7}$$

where J is the number of recorded flights of length. The form of the multinomial probability $P(j|\theta)$ depends on both the underlying continuous distribution as well as the observation protocol. We recognize and implement likelihood functions for two classes of observation protocols: 1) discretized only (2004-type likelihood from Edwards et al. (2007)) and 2) discretized and aggregated (1992-type likelihood from Edwards et al. (2007)). Both forms of the likelihood, as well as functions for creating data, are implemented in R and included as part of the supplemental materials.

S.3.1 Discretized only data (2004-type)

Data of these types consist of sequences of wet/dry indicators within short intervals of length s seconds (10 sec in the wandering albatross data from 2004). The record, R, is defined to be the number, j, of consecutive dry readings in between two wet readings (see Section S.1 for further details). We define m as the minimum interval, in seconds, that constitutes a flight (e.g., 30 sec for the data from 2004). The minimum record length that we include as a flight in our data set is $m/s + 1$, as records shorter than this can include flights that are shorter than m. Following Edwards et al. (2007)), we can write the probability of observing flights of length j as:

$$\begin{aligned} \mathrm{P}(R = j|\theta) = (1 - j) \int_{s(j-1)}^{sj} f(x;\theta)dx + (1 + j) \int_{sj}^{s(j+1)} f(x;\theta)dx \\ + \frac{1}{s}\left[\int_{s(j-1)}^{sj} xf(x;\theta)dx + \int_{sj}^{s(j+1)} xf(x;\theta)dx\right] \end{aligned} \tag{8}$$

where $f(x;\theta)$ is the pdf of the underlying flight time distribution.

Following Edwards et al. (2007)), we exclude records of length $j_{\min} = m/s$ as these can correspond to flights of less than m. However, we must account for the dry intervals

of $> m$ that we miss by excluding the records of length $j_{\min}$. The probability of obtaining a record of length $j_{\min}$ is given by

$$\mathrm{P}(R = j_{\min}|\theta) = (1 + j_{\min}) \int_{sj_{\min}}^{s(j_{\min}+1)} f(x;\theta)dx + \frac{1}{s} \int_{sj_{\min}}^{s(j_{\min}+1)} xf(x;\theta)dx \tag{9}$$

We can then obtain the full likelihood for our data by inserting Eqn. 8 into Eqn 7 and subtracting n (the number of records) times the log of 1 minus Eqn. 9:

$$\begin{aligned}\ell(\boldsymbol{\theta}|\mathbf{r}) = \sum_{j=1}^{J} d_j \Bigg(&(1-j) \int_{s(j-1)}^{sj} f(x;\theta)dx + (1+j) \int_{sj}^{s(j+1)} f(x;\theta)dx \\ &+ \frac{1}{s} \left[\int_{s(j-1)}^{sj} xf(x;\theta)dx + \int_{sj}^{s(j+1)} xf(x;\theta)dx \right] \Bigg) \\ &- n \log \left(1 - P(R = j_{\min}|\theta)\right).\end{aligned} \tag{10}$$

The pdf, $f(x;\theta)$, from any of the probability distributions described in Appendix S.2 can be used in this log likelihood. This function is then minimized numerically to obtain estimates of the parameters of the distribution.

S.3.2 Discretized and Aggregated data (1992-type)

Given memory limitations in the older types of immersion loggers, data were aggregated. The loggers deployed on wandering albatrosses in 1992 tested for saltwater immersion every three seconds, and if at least half of the tests were positive in segments of length ϵ (15 sec for the wandering albatross data from 1992), the segment was counted as wet. The segments of length ϵ were then aggregated over a larger interval s (1 hour), where only the number of ϵ-length segments that were wet in s are recorded. Thus for each interval of length s a number of segments that are wet will be an integer number on $[0; s/\epsilon]$ ($s/\epsilon = 240$ for the 1992 WALB data). Since it is impossible to discern the exact number or pattern of immersion events within the interval, the exact flight times cannot be distinguished. Thus, the record R for this case is defined to be the number, j, of consecutive completely

dry intervals in between two intervals with at least one immersion. As before we define m as the minimum interval, in seconds, that constitutes a flight (e.g., 30 sec for the data from 1992). Thus the minimum flight length is shorter than the minimum record length, which is by definition 1.

Analogous to the previous section, and following Edwards et al. (2007)) we can write the probability of obtaining a record of length j:

$$\mathrm{P}(R = j|\theta) = \int_{sj}^{s(j+1)} (x - j)f(x;\theta)dx + \int_{s(j+1)}^{s(j+2)} (2 - x + j)f(x;\theta)dx. \tag{11}$$

Similarly to the in the previous section, this equation is included in the expression for the multinomial likelihood probability and subtracted from the portion relating to $\mathrm{P}(R = 0|\theta)$ to get the full log-likelihood:

$$\ell(\boldsymbol{\theta}|\mathbf{r}) = \sum_{j=1}^{J} d_j \left(\int_{sj}^{s(j+1)} (x - j)f(x;\theta)dx + \int_{s(j+1)}^{s(j+2)} (2 - x + j)f(x;\theta)dx \right) - n \log\left(1 - \mathrm{P}(R = 0|\theta)\right). \tag{12}$$

As for the previous section, and distribution of interest can be inserted, and the log likelihood is then maximized to obtain estimates of the parameters for the underlying flight time distributions.

S.3.3 Sampling from the multinomial data distribution

Obtaining samples from the multinomial data distribution is straightforward, as for both observation methods (discretized or discretized and aggregated) a flight is the number of intervals that are fully dry.

1. Take a "true" flight time draw from a known distribution, τ.

2. Randomly choose the start time of the flight uniformly within the observation interval. That is, the start time is $t_s \overset{\text{iid}}{\sim} \mathrm{U}(0, s)$ where s is the length of the interval, for

instance 10 sec for the 2004 WALB data or 1 hour for the 1992 WALB data.

3. The length of the flight, in segments, is then calculated as $\tau_{\text{obs}} = \text{floor}((\tau - t_s)/s)$.

4. If $\tau_{\text{obs}} > m$ (i.e., of a minimum length to be counted as a flight), that flight is added to the record, R.

Acknowledgments

This project was funded by an NSF grant (PLR-1341649) to L.R.J. and S.J.R. We thank Andrew Edwards for sharing his MLE code.

Author contributions statement

L.R.J., P.H.B, R.A.P., and S.J.R. conceived the ideas; P.H.B. and S.J.R. processed raw data; L.R.J. and P.H.B. performed data simulations and fitting; L.R.J., P.H.B, R.A.P, and S.J.R. analyzed the results. All authors wrote and reviewed the manuscript.

Data access

All observational datasets used in this study are available from the Polar Data Centre at the British Antarctic Survey, Cambridge, UK (polardatacentre@bas.ac.uk). Data sets and associated simulation and analysis code have been deposited on Dryad (doi:10.5061/dryad.t1r3v).

References

A. M. Edwards, R. A. Phillips, N. W. Watkins, M. P. Freeman, E. J. Murphy, V. Afanasyev, S. V. Buldyrev, M. G. da Luz, E. P. Raposo, H. E. Stanley, et al. Revisiting lévy flight search patterns of wandering albatrosses, bumblebees and deer. *Nature*, 449(7165):1044–1048, 2007.
